# The effectiveness and cost-effectiveness of the Family Nurse Partnership home visiting programme for first time teenage mothers in England: a protocol for the Building Blocks randomised controlled trial

**DOI:** 10.1186/1471-2431-13-114

**Published:** 2013-08-06

**Authors:** Eleri Owen-Jones, Marie-Jet Bekkers, Chris C Butler, Rebecca Cannings-John, Sue Channon, Kerenza Hood, John W Gregory, Alison Kemp, Joyce Kenkre, Belen Corbacho Martin, Alan Montgomery, Gwenllian Moody, Kate E Pickett, Gerry Richardson, Zoë Roberts, Sarah Ronaldson, Julia Sanders, Eugena Stamuli, David Torgerson, Michael Robling

**Affiliations:** 1 South East Wales Trials Unit (SEWTU), School of Medicine, Cardiff University, Neuadd Meirionnydd 7th Floor, Heath Park, Cardiff, Wales CF14 4YS, UK; 2 Cochrane Institute of Primary Care and Public Health, School of Medicine, Cardiff University, Heath Park, Cardiff, Wales CF14 4YS, UK; 3 Institute of Molecular and Experimental Medicine, Cardiff University, Heath Park, Cardiff, Wales CF14 4XW, UK; 4 University of South Wales, Pontypridd, Wales CF37 1DL, UK; 5 Department of Health Sciences, University of York, Seebohm Rowntree Building, Heslington, York YO10 5DD, UK; 6 Nottingham Clinical Trials Unit, Nottingham Health Science Partners, C Floor, South Block, Queen’s Medical Centre, Nottingham NG7 2UH, UK; 7 Centre for Health Economics, University of York, Heslington, York YO10 5DD, UK

**Keywords:** Pregnancy in adolescence, Prenatal care, Maternal health, Home visiting, Birth weight, Smoking cessation, Child maltreatment, Family nurse partnership, Early years prevention, Randomised controlled trial

## Abstract

**Background:**

The Nurse Family Partnership programme was developed in the USA where it is made available to pregnant young mothers in some socially deprived geographic areas. The related Family Nurse Partnership programme was introduced in England by the Department of Health in 2006 with the aim of improving outcomes for the health, wellbeing and social circumstances of young first-time mothers and their children.

**Methods / design:**

This multi-centre individually randomised controlled trial will recruit 1600 participants from 18 Primary Care Trusts in England, United Kingdom. The trial will evaluate the effectiveness of Family Nurse Partnership programme and usual care versus usual care for nulliparous pregnant women aged 19 or under, recruited by 24 weeks gestation and followed until the child’s second birthday. Data will be collected from participants at baseline, 34-36 weeks gestation, 6, 12, 18 and 24 months following birth. Routine clinical data will be collected from maternity, primary care and hospital episodes statistics. Four primary outcomes are to be reported from the trial: birth weight; prenatal tobacco use; child emergency attendances and/or admissions within two years of birth; second pregnancy within two years of first birth.

**Discussion:**

This trial will evaluate the effectiveness and cost effectiveness of the Family Nurse Partnership in England. The findings will provide evidence on pregnancy and early childhood programme outcomes for policy makers, health professionals and potential recipients in three domains (pregnancy and birth, child health and development, and parental life course and self-sufficiency) up to the child’s second birthday.

**Trial registration:**

Trial registration number: ISRCTN 23019866

## Background

Infants born into socioeconomic deprivation are more likely to have teenage mothers, to have been exposed to cigarette smoke during pregnancy, to have poor prenatal health profiles, and are at greater risk of adverse short-term and long-term outcomes [1-5]. In 2008 there were 44,691 live births to women under the age of 20 in England and Wales [6]. Children born to teenage mothers have lower birth weights, are less likely to be breast fed, exhibit higher mortality rates, and are more likely to suffer accidents [7]. They do worse educationally, experience more emotional and behavioural problems, and are more likely to become teenage parents themselves [8].

Over one third of infants in the UK are exposed to maternal smoking and 17% of mothers in England continue to smoke throughout pregnancy [9-11]. Maternal smoking in pregnancy impairs fetal growth - the average difference in birth weight between infants born to smokers and to non-smokers is about 250g [12]. This difference increases with the amount smoked [13]. Smoking in pregnancy increases the risk of miscarriage, premature birth, Sudden Infant Death Syndrome (SIDS), emotional and behavioural problems, cognitive problems in childhood, adolescent criminal behaviour and adult violent and non-violent crime [14-17]. Maternal smoking is also negatively associated with breastfeeding initiation and duration [18].

Eight percent of babies born in the UK are of low birth weight compared to seven percent in France and Germany [19,20]. In addition to maternal smoking, risk factors for low birth weight include being a teenage mother, infection during pregnancy (causing preterm labour), family poverty and social disadvantage [21]. Consequences of having a low birth weight for gestational age or premature delivery include neurological impairment and cognitive difficulties. Adverse consequences of low birth weight on physical health and psychological distress can be found much later on in adult life [22,23].

In England, hospital and community based maternity care is provided by the National Health Service, it is universally available and free at the point of delivery. Minimum standards for maternity care are recommended by the National Institute for Clinical Excellence and include a minimum of 10 antenatal check-ups for women expecting their first baby [24,25]. Specific additional care is recommended for pregnant women under 20 and includes allocating a named midwife to provide the majority of the woman’s care, supported by direct telephone access [26]. Health Visitors (specialist community public health nurses) provide support to new mothers and their children up to the child’s fifth birthday with the level and nature of engagement depending on local resources and individual need.

Specific programmes to support the life chances of children growing up in disadvantaged circumstances have also been introduced in the United Kingdom. Sure Start Local Programmes (SSLPs) were area-based interventions for all children aged under 5, and involved local areas improving and creating services to support young families. Whilst SSLPs changed to come under the control of local authorities and run from Children’s Centres, considerable variation in delivery remained [27]. Furthermore, a more targeted programme, Sure Start Plus was piloted with the aim of reducing social exclusion associated with teenage pregnancy by providing additional community based support programmes and facilities. The pilot study of Sure Start Plus showed enhanced parenting skills, but less success in changing health damaging behaviours such as smoking [28].

The Nurse Family Partnership (NFP) programme was developed in the US to address the problems of poor birth outcomes, social exclusion, child abuse and neglect, and diminished economic self-sufficiency of socially disadvantaged younger first time mothers [29]. NFP is currently offered in 42 US states [30]. The NFP is a structured, intensive programme of home visits delivered by specially trained nurses, provided from early pregnancy until the child is two years old.

The NFP draws upon theories of human ecology, self-efficacy and human attachment [31-33]. Visits cover core content areas of personal and environmental health, life course development, maternal role, family and friends and access to health and social services. Time allocated to each content domain is prescribed by the programme but also varies over the duration of the programme. Maternal behaviour change is supported through the promotion of self-efficacy [34,35]. Education and modelling activities are included in the programme to promote sensitive and competent care giving via a strengths-based approach with the aim of reducing maltreatment. A scheduled maximum of 64 visits commence, ideally, early in the second trimester, and decrease in frequency over the first two years of the child’s life. Whilst actual number of visits received may vary by individual need, engagement, and gestational age at enrolment, minimum targets are specified to support desired programme outcomes. Programme goals are to improve pregnancy outcomes, child health and development, including a reduction in child maltreatment, and an increase in maternal self-sufficiency.

In three randomised trials in the US, the NFP programme improved prenatal health behaviours, birth outcomes, sensitive child care, reductions in child injuries, abuse and neglect, maternal life course (e.g. greater workforce participation, fewer subsequent pregnancies, reduction in welfare requirements), and child and adolescent functioning [29,36-45]. The NFP had greatest impact amongst those with low psychological resources. The cost of the programme for low-income and unmarried mothers was recovered by the child’s fourth birthday, whilst amongst married women or those of higher socioeconomic status a net financial saving has been demonstrated over a longer period [46].

NFP has been adapted for the UK where it is known as the Family Nurse Partnership (FNP) programme and is part of the Healthy Child Programme in England. Formal evaluation has demonstrated that FNP is deliverable and acceptable within the UK with some encouraging findings in terms of outcomes [47-49]. Although there is evidence of programme effectiveness in the US, there is no rigorous trial evidence for FNP in the UK, which has universal access to more comprehensive supportive care for young first-time mothers [24,25]. It is this evidence of short-term outcome effectiveness (up to the child’s second birthday) that will be generated by the Building Blocks trial.

The trial is supported by a partnership between government, research and primary and secondary care teams. In 2008, Primary Care Trusts (PCTs) in England tendered to the Department of Health to provide the FNP programme within the study context. The successful 18 PCTs subsequently employed the FNP teams to deliver the programme. The Department of Health based FNP central team will provide FNP materials, training and ongoing support to the PCT local teams and maintain a central database of FNP activity.

FNP teams are comprised of up to eight nurses, each of whom carries a maximum caseload of 25 clients, a supervisor who carries a reduced caseload, and an administrator. This is similar to the NFP programme.

### Research aim

The aims of our trial are to:

Aim 1: Evaluate the effectiveness of the FNP within three outcome domains: pregnancy and birth, child health and development, and parental life course and self-sufficiency. Four primary outcomes have been identified: birth weight, changes in prenatal tobacco use, emergency attendances and hospital admissions for the child within two years, and proportion of women with a second pregnancy within two years. Primary and secondary outcomes are listed in Tables 1 and 2.

Aim 2: Assess the incremental costs and consequences of the FNP programme compared to existing services.

Aim 3: Model possible longer term costs and effects of the FNP programme.

Aim 4: Evaluate what processes influence FNP outcomes in order to explore applicability to other settings and to optimise its future implementation.

**Table 1:** Maternal outcomes and assessment points

**Outcome (name of measure**^**1**^**)**	**Baseline**	**34-36weeks**	**Birth record**	**6 months**	**12 months**	**18 months**	**24 months**	**HES / GP**^**2**^
***Socioeconomic***								
Not in education, employment or training (NEET status) [50,51]	***x***			***x***	***x***	***x***	***x***	
Hours in formal education [50,51]				***x***	***x***	***x***	***x***	
In paid employment [50,51]	***x***			***x***	***x***	***x***	***x***	
Type of employment [50,51]	***x***			***x***	***x***	***x***	***x***	
In receipt of benefits [50,51]	***x***						***x***	
Other financial support [50,51]	***x***						***x***	
Ever been homeless (Millennium Cohort Study) [50,51]	***x***			***x***	***x***	***x***	***x***	
***Maternal health*&*well-being***								
General health status (EQ-5D) [52]	***x***	***x***		***x***	***x***	***x***	***x***	
Weight / BMI (Millennium Cohort Study) [50,51]	***x***						***x***	
Psychological distress (Kessler scale) [53]	***x***						***x***	
Depression (Whooley scale) [54]				***x***	***x***	***x***	***x***	
Postnatal depression (Edinburgh PDS) [55]				***x***				
Self-efficacy (Generalised Self-efficacy Scale) [56]	***x***			***x***	***x***	***x***	***x***	
Adaptive functioning [57,58]	***x***						***x***	
Intimate partner violence (Composite Abuse Scale) [59]							***x***	
***Health behaviour***								
**Tobacco use**^**3**^	***x***	***x***		***x***				
Smoking cessation method (if applicable) [60,61]		***x***		***x***				
Smoke in home					***x***	***x***	***x***	
Problem alcohol and drug use (CRAFFT) [62]	***x***						***x***	
Contraceptive use and method (Millennium Cohort Study) [50,51]	***x***			***x***	***x***	***x***	***x***	
***Pregnancy and birth***								
Gestation at delivery			***x***					
Place of birth (planned, actual)			***x***					
**Subsequent pregnancies**^**3**^				***x***	***x***	***x***	***x***	***x***
***Social support***								
Social support and networks (MOS Survey) [63,64]	***x***			***x***	***x***	***x***	***x***	
Family resources [65]	***x***			***x***	***x***	***x***	***x***	
Relationship quality (Golombok Rust Inventory of Marital State) [66]	***x***	***x***		***x***	***x***	***x***	***x***	
***Use of services***								
Dental care							***x***	
Antenatal care (check-ups, planned / unplanned attendances)			***x***					***x***
Primary care or secondary care attendance / admission		***x***		***x***	***x***	***x***		***x***
Additional non-health (education, social, childrens, Connexions) services				***x***	***x***	***x***	***x***	
Foster care (mother, baby, both)				***x***	***x***	***x***	***x***	

^1^ Name of validated measure (items amended or partially sourced from existing measures indicated by citation only) ^2^ For period from recruitment or birth until 24 months (mother and child); ^3^ Primary outcome domain.

**Table 2:** Parenting and child outcomes and assessment points

**Outcome (name of measure**^**1**^**)**	**Baseline**	**34-36weeks**	**Birth record**	**6 months**	**12 months**	**18 months**	**24 months**	**HES / GP**^**2**^
***Parenting beliefs, behaviour and experience***								
Anticipatory parenting (Millennium Cohort Study) [50,51]		***x***						
Infant feeding intentions		***x***						
Prenatal attachment [67]		***x***						
Parental role strain (Millennium Cohort Study) [50,51]				***x***	***x***	***x***	***x***	
Maternal-child interaction [68]							***x***	
Mother and child living apart				***x***	***x***	***x***	***x***	
***Neonatal outcomes***								
Live birth			***x***					
**Birth weight**^**3**^			***x***					
Apgar score (1, 5 mins)			***x***					
Head circumference			***x***					
Neonatal unit admission			***x***					
***Feeding*&*development***								
Breastfeeding initiation, duration				***x***	***x***	***x***	***x***	
Baby diet						***x***	***x***	
Cognitive development [69]					***x***	***x***	***x***	
Language development (Early Language Milestone Scale) [69,70]					***x***	***x***	***x***	
Child safety [71]					***x***	***x***	***x***	
***Health and use of services***								
Childcare				***x***	***x***	***x***	***x***	
Immunisations				***x***	***x***	***x***		***x***
**Emergency attendances&admissions (all cause)**^**3**^		***x***		***x***	***x***	***x***	***x***	***x***
Primary care consultation (injuries & ingestions)		***x***		***x***	***x***	***x***		***x***
Medically attended injuries & ingestions		***x***		***x***	***x***	***x***	***x***	***x***
Referral from primary care (social care, other, safeguarding)							***x***	***x***

^1^ Name of validated measure (items amended or partially sourced from existing measures indicated by citation only) ^2^ For period from recruitment or birth until 24 months (mother and child); ^3^ Primary outcome domain.

## Methods

### Trial design

This trial is an individually randomised controlled trial with a parallel economic modelling study. Trial participants ***either*** receive usual universal services ***or*** usual universal services along with regular visits from trained Family Nurses (FN) from early pregnancy (of their first child) until the child is two years old, following a prescribed programme.

### Intervention

#### FNP

Participants in the intervention arm will receive up to 64 home based visits from the FNP nurse during their pregnancy and in the two years following childbirth. Fidelity requirements on programme recruitment and delivery are specified by the developers of the US programme (Table 3).

**Table 3:** **FNP fidelity goals **[47,48,72]

**FNP fidelity goals**	**Criteria**
**Recruitment and enrolment**	• at least 60% enrolled before 16 weeks of pregnancy and 100% no later than 28 weeks
	• 100% of clients enrolled are first-time mothers
	• 100% of clients enrolled are 19 years or younger at LMP
	• 75% of eligible clients who are offered the programme are enrolled
	• each family nurse enrols 25 families (or pro rata) within 9 months of recruitment
**Attrition**	Clients leave the programme at no more than these rates:
	• cumulative programme attrition is 40% or less through to the child’s second birthday
	In detail:
	• 10% or less during the pregnancy phase
	• 20% or less during the infancy phase
	• 10% or less during toddlerhood
**Dosage**	Clients receive:
	• 80% or more of expected visits during pregnancy
	• 65% or more of expected visits during infancy
	• 60% or more of expected visits during toddlerhood
	On average, length of home visits is around 60 minutes

In the UK, the Family Nurse provides the FNP programme, alongside the usual maternity services during pregnancy and the neonatal period. From one month to two years after birth, the Family Nurse continues to provide the programme undertaking the role of the Health Visitor. Around the time of the child’s second birthday care is handed over to a Health Visitor, whose input will vary depending upon recognised need and local resources.

Family Nurses are recruited from existing registrants on the Nursing Midwifery Council of the UK, mainly from Health Visiting but also from Nursing and Midwifery. Training in delivery of the programme will be provided to the Family Nurses by the FNP central team.

#### Usual care

Participants allocated to the usual care will receive care from the local maternity services in line with usual practice. Following birth, the participants will continue to receive postnatal midwifery care and care from existing child health services available locally, including an allocated Health Visitor.

### Site and participant selection

Local consortia, comprising chiefly of individual Primary Care Trusts (PCTs), and local authorities formally applied to the Department of Health to be provider sites for FNP and expressed willingness to participate in the randomised trial. Each site was required to demonstrate sufficient local clinical need and collectively to yield sufficient trial recruits during the planned recruitment period.

The 18 selected trial sites are located in Barnsley, Berkshire East, Cornwall, Coventry, Cumbria, Derby, Hull, Lambeth, Leeds, Liverpool, Manchester, Northamptonshire, South Birmingham, South East Essex, Southwark, Sunderland, Tower Hamlets and Walsall. In most cases FNP will be delivered across the whole area covered by each PCT, but for some sites (e.g. Cornwall) availability of the programme will be partially restricted to certain parts of the PCT area.

### Local researchers

Local researchers (usually a midwife or nurse), will be employed at each trial site, and trained by the trial team prior to their site opening to recruitment and annually thereafter. Topics to be covered during the training sessions will include an overview of the trial, the FNP programme, obtaining consent and recruitment procedures. Emphasis will be placed on the assessment of the competence of potential trial participants to provide consent, explaining both arms of the trial without bias, trial outcomes, serious adverse event monitoring and reporting, and withdrawals. Later training will cover follow-up rates, retention strategies, birth related data collection, organising and conducting the 2 year home based interview, data collection from primary care records and conducting research interviews.

The role of a local researcher will be to recruit participants, maintain current contact details for participants and conduct home based interviews and assessments in their trial site. South East Wales Trials Unit, Cardiff University, in collaboration with Bristol University, University of York and University of South Wales (formerly University of Glamorgan) will implement the trial.

### Eligibility criteria

Eligibility criteria will match FNP programme enrolment criteria as closely as possible and are listed in full in Table 4. Recruits will be nulliparous, aged 19 or under, and will be recruited to the trial no later than 24 weeks gestation (and ideally within the first 15 weeks). This was to enable enrolment to the FNP (for those randomised to the intervention arm) in line with the programme fidelity requirements (60% of clients to be enrolled by 16 weeks gestation, and all by 28 weeks [47,48,72]).

**Table 4:** Participant inclusion/exclusion criteria

**Inclusion criteria**	**Exclusion criteria**
1. Women aged 19 or under (at recruitment / consent).	1. Women who at study entry, plan to have their child adopted.
2. Lives within the catchment area covered by the local FNP team.	2. Women who at study entry, plan to leave the FNP area during the time of the trial either for an extended period of time (3 months or longer) or permanently.
3. First pregnancy confirmed by health services (including those expecting multiple births) unless previous pregnancy ended in miscarriage, stillbirth or termination.	3. Women who would require a third person (translator, sign interpreter) to receive the FNP programme.
4. Recruited no later than 24 weeks gestation.	
5. Gillick competent to provide informed consent to research participation including competence in English at conversational level or higher.	

### Recruitment

Potential participants will be identified in conjunction with staff from antenatal clinics. Women may also either self-identify or be referred via other services including GP surgeries into the study. Referral pathways will be tailored to accommodate local variations in clinical practice pathways and services at each site. Trial information will be made available at local clinics (midwifery clinics, GP surgeries, Children’s Centres, Local Connexions Offices). During the study recruitment period women in study sites will only be able to enrol in the FNP programme via participation in the trial. Direct referrals to the FNP team will be passed back to the Local Researcher to discuss trial participation.

Local Researchers will contact identified potential participants to arrange a recruitment visit where they will be provided with an information pack explaining the trial. Adequate time will be given for reading the material and asking any questions. Women will be encouraged to discuss the trial with friends and family, if needed, before deciding about participation. Informed, written consent will be obtained by the local researcher.

### Randomisation

Prior to recruitment of a trial participant, the Local Researcher will confirm that the local FNP team has spare capacity, consent will be obtained and the baseline assessments completed. The Local Researchers will use a remote randomisation service (automated telephone or web) provided by Bristol Randomised Trials Collaboration (BRTC) to allocate the woman to FNP or usual care. The Local Researcher will inform the FNP team of participants allocated to receive the programme. The FNP team will then take responsibility to enrol the woman into the programme and provide the intervention. For women allocated to usual care, the woman’s community midwife will be informed. All participants’ General Practitioners will be notified of their recruitment to the trial.

A randomisation algorithm will minimise by gestation at recruitment (<16 weeks/16 weeks+), smoking status at recruitment (smoker/non-smoker) and first language/preferred language (English/non-English) for each site with a probability of 0.8. Therefore a random element is retained, further reducing predictability of allocation. The trial statisticians and economists will be blinded to treatment allocation until analyses are complete.

### Frequency and duration of follow-up to assess study outcomes

Follow-up will continue until the child’s second birthday. Following the baseline home assessment, women will be followed up by qualified and specially trained telephone interviewers at 34-36 weeks gestation, then at six months, twelve months, and eighteen months postnatally. A final home-based face-to-face interview will be conducted two years after birth. The interviewers will follow procedure-specific Standard Operating Procedures with regards to serious adverse event reporting and child safeguarding issues.

**Figure 1:**
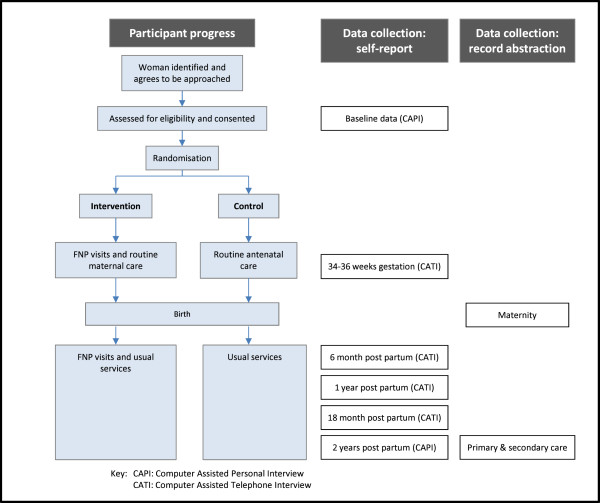
Participant flow diagram and data collection.

A urine sample for cotinine assessment will be collected at the baseline visit and also at 34-36 weeks gestation. Following birth, medical and obstetric history items, antenatal attendances, and maternal and neonatal birth outcomes will be extracted from medical records onto a Case Report Form. Data relating to the number, duration and content of Family Nurse visits to participants will be captured on a Department of Health database which the trial team will access. The timing and methods of data collection are summarised in Figure 1.

### Retention strategy

Local researchers will be the main point of contact between the trial team and trial participants and will have a key role in maintaining participant engagement with the trial. They will update participant contact details, monitor for adverse events and promote progress of the trial. The trial team will visit sites prior to initiation, and run central training days where all the local researchers get to know one another, share best-practice procedures and be trained in how to carry out subsequent trial phases.

To maintain engagement with trial participants, based on suggestions from local stakeholders including young mothers, we will use vouchers for telephone airtime, birthday cards and presents for the baby, contacts from the local researcher, and use of SMS texting. High Street vouchers to the value of £25, £25 and £40 will be given after completion of the 12, 18 and 24-month interviews respectively to acknowledge their time commitment to data collection. A first year birthday gift will be sent and a second year birthday gift will be given on completion of the two year interview. Prior notification of telephone interviews will be via SMS messages to participants. A participant website will be used to encourage engagement of participants.

### Primary outcomes

Four primary outcomes are to be reported from the trial (Tables 1 and 2):

•*Birth weight (grams)*.

•*Prenatal tobacco use:* calibrated number of cigarettes per day at 34-36 weeks gestation [73];

•*Emergency attendances and/or admissions within two years of birth*: proportion of participants’ children attending Accident and Emergency (A&E) or having an emergency admission to hospital within two years of birth.

•*Second pregnancy within two years of first birth*: proportion of women with a subsequent pregnancy within two years of first birth.

### Secondary outcomes

Secondary outcomes are shown in Tables 1 and 2. Where validated scales will be used these are named and referenced; where validated scales will be modified or items selected, these are shown with a citation only; other outcome variables will be newly derived.

### Sample size estimation

A total sample of 1418 for analysis will provide at least 90% power at the two-sided 2.5% alpha level to detect differences between the two arms of 10% (40% to 30%) in the proportion having any emergency attendance or emergency admission to hospital within two years, and of 7.5% (20% to 12.5%) in the proportion with a second pregnancy within two years. For each outcome measure, the expected improvement for the FNP arm relates to a small standardised difference (approx. 0.2 or odds ratio 0.6). This small standardised difference will enable us to detect effect towards the lower end of what we expect. We will allow for a pregnancy loss of 1.5%.

We expect to obtain follow-up data for three of the four primary outcomes on at least 90% of participants by accessing the medical records in hospital and primary care for both the mother and child. Follow-up smoking rates will be collected through the late pregnancy telephone interviews. We will therefore aim to recruit a total of 1600 pregnant women to achieve our target number for analysis, as this takes into account anticipated pregnancy loss and loss to follow up.

We have chosen to use a 2.5% alpha level to allow for multiple primary outcomes within each individual population in the trial – i.e. there are two primary outcomes for the mother (smoking and second pregnancy) and two for the baby (birth weight and emergency attendances/admissions). This gives a 5% type 1 error rate for each population.

### Stakeholder involvement and ethics

A Stakeholder Involvement and Ethics Group will be responsible for working across the trial, economic and process evaluations. We recognise that pregnant teenagers are a vulnerable group, at greater risk of social exclusion (e.g. from lower socioeconomic groups, care leavers). Consequently, various sensitive and practical issues need consideration prior to study commencement, for example, consent for those aged under 16 and procedures to be followed in the event of child safeguarding concerns. The Stakeholder Group will work to identify potential issues that may arise during the course of the trial and advise on their appropriate management. This will entail the development of a range of Stakeholder Groups to support the conduct of the trial through the various stages of development to completion.

The collaborative involvement of lay and professional people in the Stakeholder Groups will provide added value to the trial teams. Public contributors will be part of core trial management groups: Trial Steering Committee, the Stakeholder Management Group and the Independent Data Monitoring Committee. Reference groups, including young mothers, will be set up to advise on trial information materials, provide advice at the different stages of the trial, and contribute towards the design of the trial participant website.

#### Process evaluation

The process evaluation will aim to understand variations in the impact of the intervention and to contextualise findings [74]. Routine FNP monitoring data will be integrated with planned process evaluations using quantitative and qualitative methods [75].

### The process evaluation will examine

•fidelity to programme content and style of programme delivery, and to pre-set frequency and duration targets

•participants’ engagement and satisfaction with FNP

•impact of FNP roll-out as perceived by local stakeholders (FNP teams, Health Visitors, Midwives)

•wider contextual influences on programme implement-ation and outcomes

Fidelity targets for FNP visits prescribe time allocation for different content domains. In the UK, delivery of the programme is integrated with Motivational Interviewing (MI) [76] which forms part of the foundation of the FNP. Fidelity assessment will include audio-recording a sample of home visits for women at each site (n=216). Recordings will be assessed for consultation content and MI competencies using thematic content coding and a validated scale measuring adherence to Motivational Interviewing techniques [77]. Dose will be assessed by comparing FNP monitoring data against set performance metrics. Interviews with a sample of FNP clients (n=72) will assess intervention exposure; participants in the non-intervention arm of the trial will not be interviewed. Trial recruitment and retention will be assessed by examining sample characteristics and attrition rates and predictors.

Evaluation of context will focus on mapping existing universal services using questionnaires with key informants at each site and accessing national and local policy guidelines and commissioning briefs. Planned analysis of trial outcome data will explore differential programme impact across practitioners, sites and sample sub-groups.

Further contextual factors affecting implementation and potential wider roll-out will be explored in telephone interviews with FNP supervisors and focus groups with FNP teams and other local Stakeholders (Midwives, Health Visitors). Assessing contamination will form part of the structured telephone interviews with local stakeholders and the focus group discussions.

#### Health economics and modelling

Intervention effects will extend beyond the health sector, both within the timeframe of the trial and in the future. Therefore two components of work are required: one to explore the relative value of effects within the different sectors (health, social care, education, criminal justice) and another to consider the longer term extrapolation of trial results (i.e. over the lifetime of the child). To inform both of these, a literature review of economic outcomes within criminal justice and education literature will be conducted.

Valuation of effects: preference elicitation work will be undertaken in order to measure the relative values that members of the general public place on the different outcomes of the trial. Specifically, a discrete choice experiment and a best-worst scaling exercise will be conducted. This work will consider the trade-offs that people are prepared to make between benefits in the different sectors, which will inform the relative magnitude of benefits accruing to different sectors.

Economic extrapolation: the extrapolation exercise will seek to link the primary outcomes of the trial with longer term outcomes in health, education, employment and criminality extending to the childhood and adulthood of the index baby, and the mother. A literature review will be conducted to capture studies that examine the association between the short term primary outcomes, as measured in the trial, and the longer term outcomes. The inclusion of the studies will be done in stages, where cohort studies of longitudinal nature that enrolled participants at birth stage or prenatally and were conducted in the UK will be prioritised. The results of the literature review will be summarized in a narrative way and all the links will be presented in a tabulated format. The results of the review will be interpreted in light of the FNP trial results.

### Statistical analysis

#### Main analysis – primary outcomes

The primary comparative analyses will be conducted on an intention-to-treat basis, with an emphasis placed on confidence intervals for differences between the randomised arms.

Descriptive statistics of demographic and outcome measures will be used to ascertain any marked imbalance between the arms at baseline. For birth weight and prenatal tobacco use at 34-36 weeks the primary comparative analyses will employ multivariable linear regression to investigate differences between the arms, adjusting for stratification/ minimisation variables. In the case of prenatal tobacco use the calibrated number of cigarettes per day reported at baseline will also be adjusted for. The result will be presented as the (adjusted) difference in mean number of cigarettes per day between the intervention and control arms. Parameter estimates will be shown alongside 95% confidence intervals (CIs) and p-values.

For the proportion of children with at least one emergency attendance and/or admission and proportion of women with known subsequent pregnancies, logistic regression will be used adjusting for stratification/minimisation variables. Comparisons will be presented as odds ratios, 95% CIs and p-values.

Multilevel modelling will be used to allow for clustering of effect within a site and Family Nurse. Where this analysis indicates little impact of clustering on effect then the results from the single level model will be presented. Sensitivity analyses will be conducted to investigate the potential impact of missing data, making different assumptions such as ‘best’ and ‘worst’ case scenarios as well as imputation models. Where variables exhibit marked imbalance at baseline, primary analyses will be repeated and adjustments will be made to check that this does not influence findings. The same analyses will be carried out for the secondary outcomes depending on the type of data.

Appropriate interaction terms will be entered into the primary regression analyses for each of the outcomes in order to conduct pre-specified subgroup analyses. These subgroups will be defined by the Trial Management Group in advance of any analysis being started with input from the trial team and summaries of current evidence from the literature. Since the trial is powered to detect overall differences between the arms rather than interactions of this kind, the results of these exploratory analyses will be presented using confidence intervals as well as p-values.

Routine data on compliance with the FNP and usual maternity and early childhood services will be collected and utilised in understanding any outcome differences between the two arms.

#### Process evaluation analysis

To establish internal validity, a sample of audio-recorded pregnancy, infancy, and toddlerhood home visits will be compared for self-reported and independently established programme content, using descriptive statistics. Similarly, a sample of FNP practitioners will be examined on the MI specific competency of their programme delivery using the Motivational Interviewing Treatment Integrity (MITI) validated scale ratings [77]. Dose and structure of visits will be monitored and compared to pre-set targets. Recruitment and retention to the trial will be monitored on an ongoing basis. Contamination between trial arms will be established using descriptive statistics from the service mapping survey and follow-up trial questionnaires. The full trial data set will be subjected to subgroup analyses to determine potential environment influences on implementation and outcomes.

### Cost effectiveness/health economics analysis

#### Costs

For the primary analysis, the resource use data related to health care services utilised by the trial participants such as GP visits, hospital overnight stays, accident and emergency attendances will be considered. These data will be collected for both intervention and control arm from self-reported questionnaires, the NHS Hospital Episode Statistics and patient records held at GP practices. For the secondary analyses, in addition to the health care resource usage, non-health care costs will be included. These relate to services such as social assistance, housing, education and criminal justice. The data for this type of resource usage will be collected from the self-reported questionnaires.

The cost of the intervention will be assessed based on the routine data collected by the FNP nurses and input into the central Department of Health FNP database. This includes information directly related to the FNP utilisation such as the number and percentage of completed visits by the FNP nurses as well as their duration, and the number of telephone encounters with the client. In addition, the database includes information on the qualifications of the FNP nurses which will be used to assess the remuneration bracket of the nurses for the overall costing of this service.

#### Outcomes

For the economic analysis, the same primary outcomes that have been considered in the statistical analysis will be used. These will be analysed and reported as outcomes in a cost-consequences analysis or, wherever this is appropriate, in a cost-effectiveness analysis. Where data allow, these effectiveness estimates will be linked to utility scores and a cost-utility analysis will be conducted. A cost-utility analysis will be used for estimating the impact of the programme on maternal health. The outcome for this analysis is measured by calculating quality-adjusted life years (QALYs). Direct estimation of QALYs is not possible for children as they will be too young during the trial, but we will estimate the intervention impact upon the mothers using the EQ-5D. The cost-utility analysis will be based on the QALYs difference of the mothers between the two arms from 34-36 weeks gestation of the index child and up to the second birthday of that child, when the trial is completed.

#### Unit costs

Unit cost estimates will be applied to resource use data to generate individual level cost estimates. Sources of unit costs will include routine or published literature such as the latest Personal Social Services Research Unit (PSSRU) Unit Costs of Health and Social Care [78] and the latest NHS Reference Costs [79]. In addition, published sources and information from local health care providers will be sought. For the non-health related resources, unit costs will be sought from the relevant sources such as governmental websites.

#### Data analysis and presentation of the results

Two types of analyses will be conducted: a complete case analysis (CCA) and an analysis by using multiple imputation methods; both analyses will include allowance for the clustered nature of the data. In the ‘complete case analysis’ only trial participants with available total costs and primary outcomes will be included. In the multiple imputation analysis all participants with partial information on resource usage and outcomes will be included. Missing values will be imputed by using multiple imputation (MI) methods. A range of sensitivity analyses will be conducted to test the robustness of the results under different scenarios. These scenarios will capture variability in the estimates of cost and outcomes, which result from either different methods (e.g. imputation methods) or from different sources of data (e.g. unit cost data).

Incremental cost-effectiveness ratios (ICERs) will be calculated where appropriate. These demonstrate the mean difference in costs and effect between treatment strategies, i.e. the incremental cost per QALY gained will be presented. The mean differences in costs and effects between the two arms of the trial will be estimated by using a regression based approach (allowing for covariate adjustment and subgroup analyses) and the 95% confidence intervals around those will be estimated by using bias corrected and accelerated bootstrapped methods. Cost effectiveness acceptability curves (CEACs) will be generated through the use of probabilistic sensitivity analysis. The CEAC will plot the probability of the intervention being cost-effective for a range of threshold values of an additional QALY.

### Ethical and governance approval

Multi-centre approval has been granted by the Research Ethics Committee for Wales (ref. no. 09/MRE09/8). Site-specific approval has been granted at all participating Primary Care and Acute Trusts.

### Adverse event monitoring

We will apply a procedure for the monitoring and reporting of serious adverse events (SAEs). These will be reported to the trial team by the local researchers, telephone interviewers and any other member of the local clinical team. Given the anticipated high number of pregnancy related hospital attendances and admissions, SAEs will be further assessed to identify those that are clinically complex or serious.

## Discussion

The aim of the trial is to determine whether there are important, significant differences in maternal and child outcomes during pregnancy, at birth and until the child’s second birthday between pregnant teenagers who are offered FNP and to those offered usual care in England.

Whilst there is evidence for the effectiveness of NFP from the US before the child’s second birthday, the main benefits of the NFP programme have been seen later in childhood. Therefore lack of overall programme impact cannot be concluded if this trial finds a lack of effect in the pregnancy and birth domain.

Although increased birth weight is not a direct aim of FNP, there may be an impact if rates of smoking and other lifestyle factors are influenced by the programme. Due to the clinical importance of birth weight and the availability of reliable data this has been included as a primary outcome for the child. A planned subgroup analysis of birth weight will be undertaken for mothers who smoke at baseline.

The Building Blocks randomised controlled trial has been designed to estimate impacts on outcomes occurring amongst recipients of FNP compared to routine care. As benefits of the FNP can accrue following completion of the programme, the trial includes measures which could be predictive of longer-term outcomes. At the two-year follow-up assessment (at the end of the trial), further permission will be sought to contact the trial participants to enable longer term follow-up beyond the end of the current trial period.

This trial provides an example of an evaluation of government policy. This inevitably requires management of complex interactions between a government department delivering the policy, local providers of services, locally employed researchers and university based academics. With 18 trial sites covering urban and rural settings and each with unique service provision and populations, the trial team will be required to adapt procedures and pathways to meet local need, and preserve intervention fidelity whilst maintaining the integrity of the randomised trial.

The challenges in the delivery of this trial will be complex and many. In addition to the coordination required to maintain engagement in 18 PCT sites spread across major cities and some rural areas in England, the trial will also need to engage staff groups employed by 23 NHS Trusts that provide hospital based maternity care. Specific challenges related to trial setup include the recruitment and appointment of 18 locally NHS employed researchers and the identification of suitable but flexible recruitment pathways. Participants will be active in the trial for over 2 years during which time many are expected to several change their address and other contact details. To overcome such challenges, inevitable in this trial design, an age appropriate, comprehensive retention strategy will be developed, alongside usual intensive trial management procedures.

## Abbreviations

A&E: Accident and emergency; CCA: Complete case analysis; CEACs: Cost effectiveness acceptability curves; CIs: Confidence intervals; FN: Family nurse; FNP: Family nurse partnership; ICER: Incremental cost-effectiveness ratio; MI: Multiple imputation; MI: Motivational interviewing; MITI: Motivational interviewing treatment integrity; NFP: Nurse family partnership; NHS: National health service; PCT: Primary care trusts; PSSRU: Personal social services research unit; SAE: Serious adverse event; SIDS: Sudden infant death syndrome; SMS: Short message service; SSLPs: Sure start local programmes; QALYs: Quality-adjusted life years.

## Competing interests

The authors declare that they have no competing interests.

## Authors’ contributions

MR is chief investigator of the trial. MR, KH, CCB, JG, AK, JK, JS, AM, KP, DT and GR are responsible for the design of the trial and senior trial management. EOJ, JS and GM are responsible for trial coordination, supervision and data management. RCJ, AM and ZR are responsible for statistical planning and for data analysis. MJB and SC are responsible for the process evaluation. JK was responsible for the stakeholder work. GR, ES, BCM, DT and SR are responsible for the health economics. All authors read and approved the final manuscript.

## Pre-publication history

The pre-publication history for this paper can be accessed here:

http://www.biomedcentral.com/1471-2431/13/114/prepub
